# Structure, Assembly and Function of Cuticle from Mechanical Perspective with Special Focus on Perianth

**DOI:** 10.3390/ijms22084160

**Published:** 2021-04-16

**Authors:** Joanna Skrzydeł, Dorota Borowska-Wykręt, Dorota Kwiatkowska

**Affiliations:** Institute of Biology, Biotechnology and Environmental Protection, Faculty of Natural Sciences, University of Silesia in Katowice, Jagiellońska 28, 40-032 Katowice, Poland; joanna.skrzydel@gmail.com (J.S.); dorota.borowska-wykret@us.edu.pl (D.B.-W.)

**Keywords:** cuticle, perianth, petal, sepal, cuticle mechanics, cuticle folding

## Abstract

This review is devoted to the structure, assembly and function of cuticle. The topics are discussed from the mechanical perspective and whenever the data are available a special attention is paid to the cuticle of perianth organs, i.e., sepals, petals or tepals. The cuticle covering these organs is special in both its structure and function and some of these peculiarities are related to the cuticle mechanics. In particular, strengthening of the perianth surface is often provided by a folded cuticle that functionally resembles profiled plates, while on the surface of the petal epidermis of some plants, the cuticle is the only integral continuous layer. The perianth cuticle is distinguished also by those aspects of its mechanics and development that need further studies. In particular, more investigations are needed to explain the formation and maintenance of cuticle folding, which is typical for the perianth epidermis, and also to elucidate the mechanical properties and behavior of the perianth cuticle in situ. Gaps in our knowledge are partly due to technical problems caused by very small thicknesses of the perianth cuticle but modern tools may help to overcome these obstacles.

## 1. Introduction

A cuticle is a hydrophobic boundary layer on the outer surface of primary aerial organs. Its presence is a common feature of all vascular and some non-vascular plants. Attaining the capacity to form such a boundary layer was critical to the evolutionary success of early terrestrial plants [1,2]. Not surprisingly then, various topics related to the cuticle have been thoroughly investigated and reviewed. This review is devoted to the structure, assembly and function of the cuticle from a mechanical perspective. We focus on the cuticle of highly specialized organs of the flower perianth, i.e., sepals (calyx elements) and petals (corolla), or tepals (elements of the perianth not differentiated into calyx and corolla). As the majority of cuticle studies were performed on leaves and fruits, we discuss the topics first based on these studies and then refer to investigations devoted to the cuticle of the perianth, if available, or point to putative special aspects of the perianth cuticle.

## 2. Structure, Chemical Composition and Assembly of Cuticle

### 2.1. Cuticle Structure

**Figure 1:**
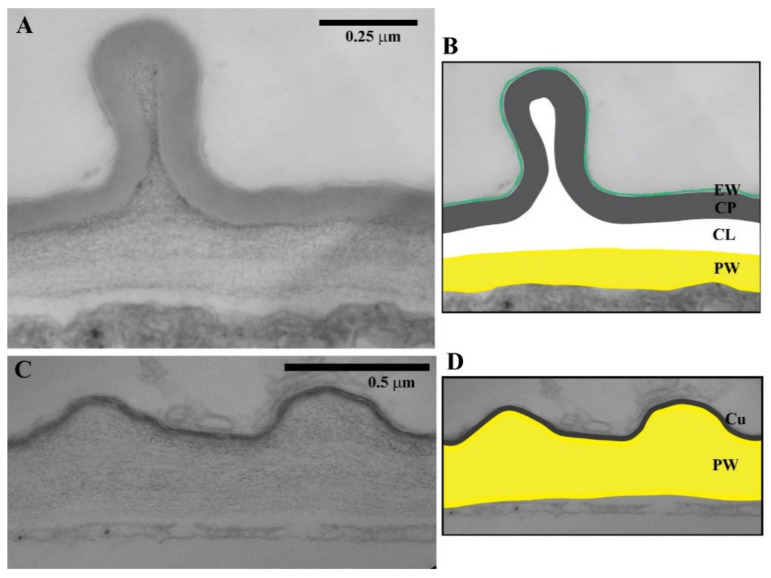
Cuticle on epidermis of arabidopsis sepal (**A**,**B**) and hypocotyl (**C**,**D**). Cross-sections of outer periclinal walls are shown in micrographs obtained using transmission electron microscopy. (**A**,**B**) Outer periclinal wall of the cell of the abaxial sepal epidermis. The cell wall layers labelled in (**B**) are: epicuticular waxes (EW); cuticle proper (CP); cuticular layer (CL); and non-cutinized primary wall (PW). The cuticle proper is folded. (**C**,**D**) Outer periclinal wall of an epidermal cell of the etiolated hypocotyl. The cuticle (Cu) overlying coves of the primary cell wall (PW) is very thin and its layers cannot be distinguished.

The structure and chemical composition of the cuticle vary among plant organs and species, and have been studied since the 19th century using a wide range of techniques [3,4]. In consequence, various interpretations of the cuticle structure and accompanying terminology have been introduced over years [3,4,5]. According to a “classic” concept, the outer periclinal wall of shoot epidermal cells (periclinal walls are those parallel to the organ surface) and the cuticle are two distinct regions [4,6]. The former is the primary wall composed mainly of polysaccharides while the latter comprises layers enriched in cutin and wax. At the interface between the primary wall and the cuticle, the pectin-rich layer is sometimes distinguished, which is continuous with middle lamellas between anticlinal walls (the walls perpendicular to the organ surface) of adjacent epidermal cells. The cuticle itself comprises three layers: the cuticular layer; cuticle proper; and epicuticular waxes (Figure 1). The cuticular layer is the innermost layer, which overlays the primary wall and consists of cellulose and other polysaccharides along with cutin and wax. The cuticular layer is covered by the cuticle proper, which is built of cutin and intracuticular waxes and is polysaccharide-free according to the “classic” definition. On the surface of the cuticle proper, i.e., at the organ–atmosphere interface, the layer of epicuticular waxes is deposited. However, recent investigations showed that this “classic” concept is a simplification. First, the structural boundary between cuticle and primary cell wall is often ambiguous. Second, in some species, polysaccharides are present across the entire cuticle thickness [4,7,8,9]. On this basis, the cuticle is now interpreted as the integral part of outer periclinal walls rather than a distinct region [10] and is referred to as a lipidized heterogeneous outer region of the cell wall [4].

In this review, we keep in mind the simplifications of the “classic” concept but nevertheless we use the classic terminology because surface walls of perianth epidermis often exhibit distinct layers, i.e., the primary wall, cuticular layer and cuticle proper (Figure 1A,B), while the presence of pectins in the cuticle proper of perianth organs has not been confirmed, at least for the petals of arabidopsis (*Arabidopsis thaliana*) [11].

### 2.2. Chemical Composition of Cuticle

Fundamental questions on the chemical composition of the cuticle have been reviewed comprehensively (see recent reviews [2,4,10]). We will thus address these topics only briefly. 

Cuticle is built of cutin, waxes and polysaccharides. The hydrophobic biopolymer cutin is a polyester of hydroxy and/or epoxy fatty acids, with the addition of a relatively low amount of glycerol, and sometimes phenolics. In some cuticles, besides cutin there is also another highly aliphatic polymer fraction, referred to as cutan or non-ester cutin. This fraction is chemically highly resistant, i.e., it remains insoluble after ester-breaking depolymerization unlike the typical cutin [4,12]. The occurrence of such fraction has been postulated to be a drought-adaptation [13]. Cutin and cutan form a scaffold (we choose the term “scaffold” rather than “matrix” because the latter often refers to the non-cellulosic polysaccharide fraction of the primary cell wall) that is impregnated with intracuticular waxes and polysaccharides [2,9]. Cuticular waxes are hydrophobic mixtures of various derivatives of mostly aliphatic, very long-chain fatty acids, with the addition of triterpenoids or phenylpropanoids [14,15]. Besides the waxes, cellulose, hemicellulose and pectins are also present in the cuticle at various concentrations, often throughout the entire cuticle thickness [8,9]. Specific modifications are known for such cutin-embedded polysaccharides. In particular, a high degree of pectin esterification, low ramification of rhamnogalacturonan and high crystallinity of cellulose were reported for the tomato (*Solanum lycopersicon*) fruit cuticle [9]. However, the fine structure of cutin-embedded polysaccharides in other species awaits further investigations. 

The composition of the cuticle varies between plant species [16], organs [17,18], epidermal cell types [19], and changes during organ development [18,20,21,22]. On the other hand, environmental factors, both biotic and abiotic, may affect the cutin and wax load as well as the composition, especially that of waxes [23,24,25,26,27,28,29,30,31]. 

Arabidopsis, the model species in plant molecular and developmental biology, is not a perfect model for studies of leaf cuticle. Namely, being a mesophyte of a short lifetime and small size, arabidopsis has a very thin cuticle which poses technical problems for cuticle isolation and investigations of its composition and physical properties [32]. Moreover, cutin synthesized by the arabidopsis leaf epidermis is rather unusual in that it closely resembles suberin, another plant lipophilic polymer that is typical for secondary rather than primary protective tissues [33,34,35]. However, unlike leaves, arabidopsis flowers produce cutin which is similar to other plant species [36,37,38].

### 2.3. Assembly of Cuticle on Organ Surface

Early in an organ development, a so-called procuticle is formed that is a thin initial layer of cuticle [5]. Starting from this moment, a new material is continuously added to the existing cuticle. Although the process from the biosynthesis of cutin and wax precursors to final assembly of the cuticle on the epidermis surface has been studied extensively, lots of problems still await elucidation, which was discussed in a number of exhaustive reviews [2,10,14,15,38,39,40]. A lot is known also about the biosynthesis and assembly of polysaccharides building primary cell walls [41,42,43,44,45,46,47,48] but much less on cutin-embedded polysaccharides [9].

#### 2.3.1. Transfer of Cutin and Wax Precursors from Protoplast to Their Destination in Cell Wall

It is well-documented that cuticle precursors, i.e., cutin monomers and wax components, are synthesized in the protoplast and released to the cell wall while the cutin polymerization and final assembly of cuticle take place in muro (inside the wall or on its surface). Thus, at first, the cuticle precursors have to traverse the plasma membrane. This process is facilitated by plasma-membrane-localized ATP binding cassette (ABC) transporters [10,14,15,49,50]. After passing the plasma membrane, cuticle precursors move to their final destination in the cell wall. A number of mechanisms were proposed for this process. The movement of cutin and wax precursors within the primary wall can be assisted by extra-cellular lipid transfer proteins (LTPs) [10,14,50,51]. Alternatives are mechanisms of non-assisted diffusion, which may be facilitated by a number of physicochemical phenomena, such as the behavior of cutin and wax precursors in the aqueous and polysaccharide-rich environment of the primary wall, or in unique environments at interfaces between water, polysaccharide, wax, and air [38]. Moreover, being compounds of low solubility, i.e., low surface energy, the precursors may spontaneously migrate via the cell wall toward the epicuticular wax layer so that the Gibbs free energy decreases [4]. Finally, the cutin and wax precursors may move via relatively hydrophobic subdomains existing within the polysaccharide cell wall, which presumably are the subdomains enriched with highly methyl-esterified pectins and glycine rich proteins [15].

#### 2.3.2. Final Assembly of Cuticle

The fundamental process in cuticle assembly is the polymerization of cutin, i.e., the esterification of its mono- or oligomers at their destination in muro. The esterification is driven by extracellular enzymes that are present in the cell wall [2,10,40]. In parallel to the enzyme-driven esterification, a self-assembly of cutin has been postulated, although this process has been shown only in vitro. For example, carboxylic acids and hydroxyl derivatives form a 2D monolayer on mica. In such a monolayer, monomer molecules are bridged via hydrogen bonds while the structure of monolayer depends on the number and position of hydroxyl groups. It is suggested that the monolayer assembly may contribute to procuticle formation prior to the esterification [52]. Furthermore, the spontaneous self-esterification that occurs upon in vitro self-assembly on the mica surface was shown specifically for one of the main cutin monomers. Molecules of this monomer formed a multilayered pattern in which parallel, nearly vertical molecules were arranged in individual layers [53]. Noteworthy, if cutin were built of regularly arranged monomer molecules in vivo, the cutin-made scaffold of the cuticle would be structurally anisotropic. However, wax components also self-assemble into the cuticle, making up the intracuticular waxes [15]. This amorphous mixture of lipids that impregnates the cutin scaffold also affects the structural properties of the cuticle. 

Another fraction of cuticular waxes self-assemble on the cuticle surface and form the epicuticular wax layer. The chemical composition of this fraction differs from the intracuticular wax fraction. The difference presumably results from the spontaneous partitioning of wax constituents due to their different physicochemical properties, or interactions of some of the constituents with polymers that are present only in the intracuticular wall region [54]. The in vitro recrystallization of waxes, isolated from cuticle surfaces of various species, leads to the formation of wax morphologies similar to those observed in vivo. Thus, the in vivo self-assembly of waxes likely involves the crystallization. The morphology of epicuticular waxes depends on their chemical composition and is affected by the physicochemical characteristic of the underlying cuticle [55].

#### 2.3.3. Cutinsomes

Some authors postulate the involvement of so-called cutinsomes in the cuticle formation. These are lipid nanoparticles formed by the self-assembly of cutin monomers in water or polar aqueous solutions [40,56,57]. Lipid nanoparticles that were observed in the cytoplasm and in cell walls were referred to as cutinsomes. Based on in vitro studies, it was postulated that cutinsomes create a physicochemical environment facilitating the polymerization of cutin monomers with no contribution of enzymes. It has been shown, also in vitro, that cutinsomes spontaneously form an amorphous cuticle-like film. The aggregation and fusion of cutinsomes on young wall surface may than be an alternative mechanism of cuticle formation in vivo, in particular as the first step of procuticle formation. Accordingly, cutinsome-based cutin assembly and enzyme-driven polymerization may function in sequence and contribute to various extents to cuticle formation at consecutive stages of organ development, as proposed for tomato fruit [58]. However, the contribution of cutinsome-based assembly in the formation of the procuticle and cuticle need to be further investigated [40,57].

#### 2.3.4. Addition of New Material to Cuticle: Superimposition and Intussusception

Starting from the establishment of the procuticle, new material is continuously added to the existing cuticle such that its continuity and stage-specific thickness are maintained despite the expansion of the organ surface while after cessation of growth, an adequate thickness is attained (see, e.g., [22,33]). Accordingly, the rate of cuticle deposition changes during the organ growth and the actual cuticle thickness results from a dynamic interplay between the deposition rate and the expansion rate of the underlying cell wall. In leaves and fleshy fruits, rates of cuticle deposition per unit surface area are usually maximal during the early developmental stages, leading to an increase in the cuticle thickness. Later during development, different relations between deposition and expansion rates may lead either to an increase or decrease in the cuticle thickness, depending on the organ and species [59]. A dynamic balance between cuticle deposition and cell wall expansion is also crucial for the formation of cuticular ridges, which are typical for the perianth surface [60]. A fundamental but yet unsolved problem related to the maintenance of the adequate thickness of the cuticle is how the cuticle thickness is “sensed” by the cell. Physical cues, such as mechanical stress, might be involved in this process. In a living cell, where turgor pressure leads to a tensile force acting in the cell wall plane, the in-plane stress is related to the wall geometry and thickness of the wall layer under consideration.

In general, the maintenance or increase in the cuticle thickness during the expansion of the underlying wall as well as an increase in cuticle thickness after the wall expansion ceases, involves an increase in cuticle volume due to addition of new material. Thus, another question that arises is about a mode by which the new material is added. In principle, two mechanisms of new material addition may contribute to the increase in the cuticle volume: a superimposition and/or intussusception-like mechanism. The superimposition of new material layers on the protoplast-facing surface of the existing cuticle seems to be a direct mechanism to control the cuticle thickness. It would facilitate both the maintenance of the thickness of the cuticle, which thins due to cell wall expansion, and the increase in the cuticle thickness after the wall expansion ceases. The intussusception-like mechanism, in which new material is inserted within the existing cuticle [61,62], could in turn lead to the expansion of the existing cuticle layer that is not accompanied by any change in its thickness. 

It has been postulated that during the tip growth of pollen tubes, intussusception is involved in the secretion of pectins and their subsequent insertion into the wall matrix. Consequently, this process leads to the growth of cell wall by loosening the existing cross-links between the matrix components, decreasing the matrix viscosity, and finally relaxing tensile stress in the wall plane [61]. The intussusception-like mechanism might as well explain the addition of new material to the cuticle. The tensile in-plane stress, which is a prerequisite for the intussusception [62], is expected in the cuticle [63,64]. Moreover, an indirect support of the intussusception-like mechanism comes from investigations of waxes in vivo. The deposition of new epicuticular waxes starts immediately after their mechanical removal from the surface of still expanding young leaves [65]. It implies that wax precursors, which are involved in this regeneration process, likely penetrate the existing cuticle layer. Similar behavior may apply also to cutin monomers. Therefore, it cannot be excluded that analogous to intussusception in pollen tube walls, new cutin monomers may penetrate the cuticle and as a result of physicochemical interactions between the existing cutin polymer and the new material, the tensile stress in the cuticle plane may be diminished, eventually contributing to the irreversible cuticle expansion.

## 3. Sculpture of Epidermal Surface—Its Variation and Origin

### 3.1. Sculpture of Perianth Surface Is Complex and Hierarchical

**Figure 2:**
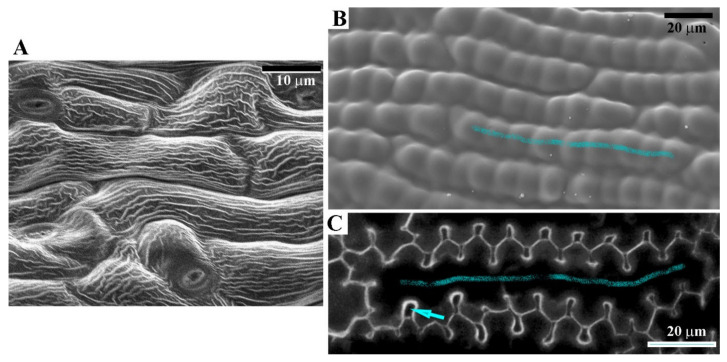
Epidermis of arabidopsis sepal (**A**) and pimpernel (*Anagallis grandiflora*) petal (**B**,**C**). (**A**) Micrograph of the abaxial surface of the mature arabidopsis sepal obtained using scanning electron microscopy. Note cuticular ridges which are formed by folds of the cuticle proper, shown in Figure 1A. (**B,C**) Petal epidermis of pimpernel. (**B**) Micrograph of the mature petal surface obtained using scanning electron microscopy. An exemplary cell is marked by a blue line. Note numerous papillae on individual cells. The papillate outer periclinal walls are covered with a continuous cuticle layer. (**C**) Anticlinal cell walls are shown in the optical section through these walls obtained using confocal laser scanning microscopy. An exemplary cell is marked by a blue line. Note numerous intercalary gas spaces at contacts between adjacent cells (arrow points to an exemplary space). In (**B**), the intercalary spaces are not visible because they are covered by a continuous cuticle.

Aerial plant organs display a variation in surface sculpture (Figure 2), the detailed classification of which is used, for example, in systematic anatomy [3]. The surface sculpture comprises the geometry of epidermal cells (cellular scale sculpture) and the sculpture of the cuticle (subcellular scale), which combines a pattern formed by epicuticular waxes overlaid on the variously shaped surface of an underlying cuticle. The sculpture of the epidermis surface is thus hierarchical [66]. 

In case of perianth organs, especially petals, the nature provides examples of especially complex surface sculptures. At the subcellular scale, the cuticle surface is often folded and forms ridges (striae) or warts [5,67,68]. At the cellular scale, the entire outer periclinal walls of the epidermis are often papillate, i.e., either individual cells have a conical–papillate shape or more than one papilla are formed on an individual cell surface (Figure 2B) [69,70]. An interesting example of the complex hierarchical sculpture is the sculpture of so-called spots located in the proximal part of the ray florets of beetle daisy (*Gorteria diffusa*) [71]. The spot is a three-dimensional elaboration of the petal epidermis composed of specialized cell types, i.e., highlight cells, interior cells, multicellular papillae, and regular pavement cells. At the tissue scale, these cells form a supracellular pattern: the highlight cells are surrounded by interior cells, which in turn are surrounded on three sides by multicellular papillae and on one side by the regular pavement cells. The cells differ profoundly in shape and the cuticle sculpture. The highlight cells are flat and covered by smooth cuticle, the interior cells are convex and their cuticle forms a prominent central ridge with deep lateral striations, while sharp striations are present on regular pavement cells. Apparently then, not only the cell morphogenesis but also the cuticle development differ between adjacent epidermal cells. 

At the subcellular scale, the sculpture of cuticle originates generally from the morphology of epicuticular waxes and the geometry of the underlying cuticle proper, the surface of which can be smooth or folded. Morphologies of epicuticular waxes are diverse and related to the chemical composition [29,55]. The epicuticular waxes may take the form of 2D and 3D structures. The 2D wax film covers the underlying cuticle proper and can be penetrated and overgrown by a 3D network of wax crystals [65]. The folding of cuticle may have different origin. In some cases, the cuticle folding results directly from the shape of the underlying cell wall, such as the cuticle of arabidopsis hypocotyl (Figure 1C,D), or from the sub-cuticular depositions of mineral crystals, e.g., silicon-oxide crystals in horsetail (*Equisetum arvensis*) [65,72]. An entirely different case is the folding of the cuticle itself, which is not a direct result of the shape of underlying structures [55], such as in the case of the cuticle on the abaxial epidermis of arabidopsis sepal (Figure 1A,B). In such cases, the space between the folded cuticle and the smooth wall is filled mainly by pectins [8,55]. The folded cuticle may form ridges arranged in a more or less ordered pattern (Figure 2A).

### 3.2. Empirical Studies and Modelling Suggest That Cuticle Folds Itself Due to Buckling

The first hypothesis explaining the folding of the cuticle itself was put forward by Martens [73], who postulated that wrinkling of the epidermal cell surface results from the oversecretion of cutin. Empirical study of development of cuticular ridges on the abaxial epidermis of arabidopsis sepals supports the role of cutin oversecretion in the cuticle folding mechanism [60,74]. The initiation and maintenance of ridges on the sepal surface are temporarily and spatially related to the differentiation of epidermal cells and cutin deposition. The ridge initiation progresses basipetally, i.e., from the sepal tip to the base, and delineates the moving frontier of the ridge-covered surface [60]. This frontier precedes slightly the frontier of cessation of cell divisions and expansion, which moves in the same direction. On the other hand, the initiation of ridges follows the expression of *CUTIN SYNTHASE 2* (*CUS2*), the encoding enzyme involved in cutin polymerization in muro. The ridges disappear when both *CUS2* and *CUS1* are silenced [75]. The latter gene is expressed earlier in sepal development. It means that ridges are initiated only when the secretion of cutin continues after the growth of the underlying cell wall slows down. In mechanical terms, the ridges are initiated because such oversecretion of cutin induces the mechanical instability of the cuticle and leads to buckling. A prerequisite for buckling is a compressive in-plane stress that acts in the smooth cuticle proper prior to buckling. The mechanism of the generation of such compressive stress needs further studies. The generation of such the stress could be related to intussusception (see Section 2.3.4). For example, if the expansion of the cuticle proper were somehow restricted, the intussusception of cutin precursors into the cuticle proper followed by the enzyme-driven incorporation of new cutin material into the existing scaffold could generate compression. The compressive stress would be anisotropic, i.e., maximal in the cuticle plane, as long as the scaffold were anisotropically reinforced. The importance of cutin production in cuticle folding is supported by lack of ridges on the sepal surface also in other arabidopsis mutants, such as *cyp77a6* and *defective in cuticular ridges* (*dcr*), in which genes encoding cytosol-located enzymes involved in cutin biosynthesis are affected [36,60]. Compressive in-plane stress could possibly also be explained by intussusception with waxes and/or polysaccharides. If these components were swelling after penetration into a cutin scaffold, the compressive stress would be generated. The occurrence of intussusception and the swelling of cuticle components is only speculative. However, an influence of intracuticular waxes on stress/strain distribution has been shown for cuticle membranes of tomato and cherry fruit [64,76]. 

In a single *cus2* arabidopsis mutant, ridges are initiated on the sepal surface but disappear progressively later during the sepal growth [60]. Thus, *CUS2* function is necessary for the expansion of the already folded cuticle proper of the growing sepal. The intussusception-like mechanism combined with wax or polysaccharide swelling could contribute to such expansion. In principle, such a shaped cuticle cannot be under tensile stress caused by the growth of the underlying wall layers. It implies that a mechanism exists of the expansion of such a non-stretched cuticle. 

The oversecretion of cuticle precursors is significant in computational models which show that the cuticle folds because it undergoes mechanical buckling [77,78]. Antoniou Kourounioti et al. [77] modelled the compressive in-plane stress as a result of mismatch (“competition”) between the transversely isotropic (isotropic within a wall layer [79]) deposition of cuticle and anisotropic growth of the underlying cell wall. This model is based on the following assumptions. First, the cuticle is incompressible, nonlinearly elastic, and it adheres tightly to the stiffer underlying cell wall. Second, anticlinal compressive stress (acting in the direction perpendicular to the cuticle surface) appears in the cuticle in response to the growth of the underlying wall. This anticlinal compression in the cuticle is likely due to the polymerization of cutin in muro, which is involved in the maintenance of cuticle thickness. The pattern of cuticular ridges on the epidermal surface reflects the distribution of in-plane stress in the cuticle before buckling. In general, this in-plane cuticle stress depends on the growth anisotropy of the underlying cell wall (related to the cell wall geometry) and rates of cutin deposition, which range from under- to oversecretion. For instance, if cutin is oversecreted on a surface of a rectangular cell, which grows anisotropically with the direction of maximal growth along the long cell axis, in-plane compression is generated in the cuticle in the direction orthogonal to the long cell axis and tension parallel to this axis. This results in the formation of the regular pattern of ridges arranged parallel to the long cell axis. A disordered pattern of cuticular ridges results in turn from biaxial in-plane compression prior to buckling. Such compression could be caused by cutin oversecretion and the nearly isotropic expansion of the underlying wall. A possible explanation of a smooth cuticle is no oversecretion combined with the isotropic expansion of the cell wall. 

The mismatch between the expansion of tightly connected layers of the epidermal cell wall is the basic assumption also in the model by Huang et al. [78]. There may be a misunderstanding concerning the structure of epidermal cell walls in this investigation, as petal surface striation is interpreted as a pattern formed by the folding of the epicuticular wax layer instead of the folding of the entire cuticle proper described by other authors [55,60,77]. Nevertheless, using the finite element model, Huang et al. [78] showed that patterns similar to those observed on petals can originate from surface buckling. In this model, however, the stiff film (referred to by the authors as epicuticular waxes) is tightly attached to the compliant substrate (the underlying cuticle and cell wall). The film expands isotropically while the growth of the substrate is anisotropic. This leads to the generation of compressive stress in the film plane, which results in buckling. Despite different model assumptions, the direction of maximal growth of the cell wall impacts on the pattern of buckling such that ridges are arranged parallel to the direction of maximal growth, like in the previously described model [77]. Therefore, the empirical quantification of the cuticle folding pattern, the growth anisotropy of epidermal cell walls, and accompanying cutin and wax deposition, combined with measurements of the mechanical properties of wall layers for the same cells, will be necessary to verify which of the models described above applies to the cuticle folding under consideration [77].

## 4. Cuticle Functions—General or Specific to Perianth Epidermis

Being located at the interface between aerial plant organs and the surrounding environment, the cuticle has to serve multiple functions (Table 1). In general, it prevents water loss, reduces the temperature of the organ surface [80], protects against abiotic factors, such as UV light [81], frost [82], and mechanical injury [83], or biotic factors, i.e., pathogens [82,84]. The cuticle also plays an important role in plant development contributing to communication between cells [80,82] and preventing fusions between organs at early developmental stages [82,85]. In the perianth, before the flower bud opens, the abaxial epidermis of sepals or tepals constitutes the outermost surface of young organs. Thus, a mainly protective role of the perianth cuticle is expected at this stage of flower development, similar to the cuticle of the stem and leaf epidermis. When the flower opens (at blossom) the protective role may become less important. Then, the sepals are sometimes shed while petal or tepal surfaces perform the functions that are flower-specific (see the list of cuticle functions specific for flower organs in comparison with leaves and fruits in Table 1).

Relations between the function of the cuticle and its chemical composition and structure are complex, as exemplified by the regulation of water permeability by the cuticle. For example, an increase in cuticle permeability was observed in arabidopsis cutin mutants *cyp77a6, gpat6* [86] while tomato mutants *cutin deficient 2* (*cd2*) and *cd3*, with strongly reduced cutin level, showed only a minor increase in the rate of water loss [87]. On the other hand, comparative studies of the water permeability of cuticles of diverse species have not indicated any correlation between the permeability and thickness of the cuticle or the amount of wax [88] while the chemical composition of waxes affects the cuticular transpiration of tomato fruit [89].

Sometimes, functions that have to be performed by the organ surface seem opposite, such as the facilitation of pollinator insect attachment to the flower surface [90] and the simultaneous prevention of attachment of nectar-robbers or sack-sucking insects [91,92]. Opposite functions are sometimes performed also by the cuticle covering different regions of the same organ. In maize (*Zea mays*) leaves, the cuticle contributes to surface stiffness and prevents dehydration of the epidermis but locally, on the surface of bulliform cells, it facilitates both strong surface deformation and dehydration [93]. In pine (*Pinus sylvestris*), the cuticle facilitates water uptake at the bases of needles, which are protected by surrounding scales, while the cuticle covering exposed needle portions is strongly hydrophobic and causes the movement of water droplets toward the needle bases [94]. Opposite functions are performed also by cuticles of different organs of the same plant. For example, the cuticle prevents young growing organs from fusion [82,85] while on the other hand, it facilitates close interactions between pistil and pollen grains [80,85].

## 5. Cuticle Mechanics and Mechanics-Related Functions

In mesophytes, such as arabidopsis, measurements of mechanical properties of the cuticle are technically challenging because their cuticles are very thin and difficult to handle after isolation [32]. In consequence, despite the availability of cuticle mutants in arabidopsis or other dicot mesophytes such as snapdragon (*Antirrhinum majus*), the majority of investigations dedicated to the cuticle mechanics were performed on cuticular membranes isolated from fruits, such as tomato, sweet cherry (*Prunus avium*) or apple (*Malus domestica*), and sclerophyllous leaves, the cuticles of which are much thicker than those of mesophyte leaves or perianths. The cuticles of fruits and sclerophyllous leaves are special in that the cuticle proper and cuticular layers are usually not distinct regions and both are included in isolated cuticular membranes. Moreover, the cuticle forms wedge-shaped protrusions (pegs) which usually reach deep into anticlinal walls, such as in sweet cherry [112,113], some apple cultivars [133] and sclerophyllous leaves [63]. As the pegs are also a part of isolated cuticular membranes, the membrane thickness is not uniform. In addition, in fruits of tomato and many apple cultivars, cutin and wax impregnate not only the outer periclinal walls of epidermal cells but also deeper located walls, sometimes even walls of subepidermal cells. In consequence, the isolated cuticular membranes comprise a few layers of cutinized walls [83,134]. On the contrary, the surface of petals and sepals is usually covered by a cuticle proper that is a distinct continuous layer of nearly uniform thickness and chemical composition. Importantly, the pattern formed by the folded cuticle is often continuous at the supracellular scale (see, e.g., [68]). It suggests that in mechanical terms, the cuticle on such an organ surface operates as one entity at the supracellular scale, i.e., when the tissue is under mechanical stress, the cuticle can transfer a slight change of supracellular mechanical stress (tensile or compressive) across neighboring cells. The cuticle proper isolated from the perianth epidermis would thus be an ideal object for mechanical tests. However, to our best knowledge, mechanical properties of the perianth cuticle have not yet been investigated using direct measurement methods. Physical properties of the arabidopsis petal cuticle were examined only in terms of petal surface adhesion to a glass surface, which were shown to depend on the surface sculpture, i.e., cuticle folding and cell shape [11]. Other comprehensive studies were devoted to the interaction of the perianth surface of other plant species with water [135] and insects [90,92]. 

Cuticle mechanics have been thoroughly discussed in excellent reviews dedicated mainly to fruit and leaf cuticles [56,59]. Here, we address selected topics related to cuticle mechanics and function in general, pointing to specific problems related to the perianth cuticle.

### 5.1. In Biomechanical Terms Cuticle Is a Layer of Composite Material Showing Viscoelastic Behavior

From the perspective of material science, the cuticle is made of a composite material [38], in which waxes and polysaccharides fill spaces between a cutin scaffold. This composite nature of material affects the mechanical behavior of the cuticle. Tensile tests with cuticular membranes show that the relationship between force (stress) and strain is typical for viscoelastic material [56,59,136]. Such material responds to externally applied forces by large strains that increase over time and can be slowly recovered, either totally or partially, when the force drops to zero. Under a constant external force, the magnitude of strain of a viscoelastic material changes in time. In other words, viscoelastic materials show a time-dependent mechanical behavior [1,137]. The force–strain diagram for cuticular membranes usually comprises two parts (a biphasic diagram). The first part, with a higher slope, is linear and dominated by an elastic strain. The second part, which represents stronger deformations, usually has a lower slope and is a combination of viscoelastic and plastic strains [56,59]. 

Other mechanical phenomenon reported for cuticular membranes is strain–hardening [56,134]. Cyclic tensile tests show that under increasing force, the cuticular membranes harden which is manifested by an increasing Young’s modulus. The strain–hardening might be a consequence of the reorientation of fibrillar components of the cuticle, such that they become aligned in the direction of the applied tensile force. Despite the presence of fibrillar components and birefringence that was reported for cuticular membranes [138], the cuticle behaves as an isotropic material [56]. 

Another important observation is that the Young’s modulus of the cuticle and cuticle strength (tensile stress at breakage) depend on relative humidity and temperature within their physiological ranges [56,59,136,139]. Namely, both the rigidity and strength of the cuticle decrease with hydration or increasing temperature. Water acts on the cuticle as a plasticizer, most likely by decreasing the viscosity of the cutin and polysaccharide fraction of the cuticle. Analysis of the hydration effect on dewaxed cuticular membranes showed the hydration-driven enhancement of the local mobility of cutin scaffold elements (acyl chain segments) [140]. The temperature effect in turn is related to temperature-dependent glass transitions of cutin. At the glass transition temperature, which is circa 23 °C for cutin and 33 °C for cutan, a reversible transition takes place between a rigid behavior and rubber-like state [141].

### 5.2. Mechanical Properties of Cuticle Depend on Its Structure and Chemical Composition and Are Changing during Organ Development

Mechanical properties of the cuticle depend on a combined effect of its chemical composition and ultrastructure. The characterization of cuticle-related mutants and the comparison between isolated cuticular membranes from which specific components were extracted, show that different cuticle components are responsible for various mechanical functions. The cutin scaffold alone, characterized by a low Young’s modulus and high maximal strain, is responsible for the viscoelastic behavior of the cuticle [56]. Intracuticular waxes [56,87,136,142,143], which fill spaces between the cutin scaffold, as well as polysaccharides (cellulose, pectin and cellulose) or phenolic compounds, which are typically present in cuticle of tomato, increase the Young’s modulus and stiffness of cuticle. Waxes also reduce the susceptibility of the cuticle to fracture [136]. The postulated explanation of this effect is that waxes act as fillers that reduce the mobility and flexibility of the cutin scaffold [59,136]. 

The mechanical properties of cuticle vary between species and organs similar to the variation of cuticle composition and structure. Wide ranges of Young’s modulus and of the maximal stress and strain at breakage were reported for cuticles of various plant species, with up to tenfold differences between minimal and maximal values of these parameters [59]. As both the composition and structure of cuticle change during the organ development, physical properties of the cuticle also change during the development of individual organs (reviewed in [59]). Due to its viscoelastic behavior, the cuticle can serve as a barrier and provide a mechanical protection in young growing organs, and at the same time it does not restrain the organ growth [56]. On the other hand, expansion of the organ surface may pose a challenge to cuticle integrity, the effect which is most pronounced in fast growing fleshy fruits [143]. Generally, during organ maturation, both the Young’s modulus and strength (the maximal stress at breakage) of the cuticle increase, while the maximal strain decreases [59]. The changes in the mechanical properties of the cuticle during development are correlated with changes in chemical composition. In mangrove (*Sonneratia alba*), cuticle membranes isolated from young leaves have low breaking stress, low Young’s modulus and are viscoelastic, while at maturation the membranes strengthen, stiffen (Young’s modulus and breaking stress increase) and elastic behavior starts to dominate. These changes are correlated with the increased deposition of cutan and polysaccharides [20]. 

Mechanical properties of the cuticle are likely related also to the adaptation of plants to different environmental conditions [63]. For example, the cuticle of *Yucca*, the species adapted to desert environment, shows a high contribution of elastic behavior (mostly linear stress–strain curves) and its fracture surface at breakage under tensile stress is smooth, resembling hard and brittle polymers. The opposite behavior is that of the leaf cuticle of ivy (*Hedera helix*), growing in temperate climate, the stress–strain curves and fracture surface of which are typical for viscoelastic and ductile material.

### 5.3. Cuticle Contributes to Stiff Faces of Plant Organs That Exhibit a Sandwich-Like Structure

From the engineering perspective, a lamina of a typical plant leaf is a sandwich-like structure, i.e., it comprises two faces (adaxial and abaxial), characterized by a relatively high Young’s modulus, which are separated by a low modulus core [144]. Such a design is advantageous for thin structures that have to be stiff in bending. The surface stiffness of shell-like structures, including the sandwich-like ones, has been extensively studied by biophysicists [145]. The cuticle plays an important role in such the sandwich-like organs because of its mechanical strength [59] and because cuticle layers add to the overall thickness of the outer periclinal walls. Accordingly, the modulus of the epidermis is strongly correlated with the fraction occupied by the cuticle and an underlying cell wall, and it is postulated that the effective thickness of the epidermis is only that of the outer periclinal cell wall with the cuticle [144]. 

A similar sandwich-like design applies to sepals and petals, which are thin plate-shaped organs with the distribution of tissues, which differ in Young’s modulus, similar to leaf blades. The role of the cuticle in the sandwich-like construction is the most prominent in petals of plant species in which intercalary air spaces are present between epidermal cells, such as pimpernel (*Anagallis*; Figure 2C), *Pelargonium* or flax (*Linum*) [70,146,147]. Such intercalary air spaces are delineated by anticlinal walls of two neighboring epidermal cells, i.e., they form gaps in the epidermal cell layer. In consequence, the cuticle is the only continuous layer covering the petal surface (Figure 2B,C) and it alone ensures organ integrity and provides surface stiffness. 

It has to be kept in mind that mechanical strengthening of the organ surface provided by a folded cuticle typical for the perianth surface is not the same as the strengthening by a smooth cuticle. The main difference is that in a folded cuticle proper (in the case of surfaces with cuticular ridges) is not under tension and thus does not bear and transmit loads like the stretched smooth cuticle, e.g., covering the surface of sclerophyllous leaves. Cuticle folding can nevertheless be an advantage in mechanical terms: a folded stiff film (cuticle proper) attached to a soft medium (pectin-rich wall region) may provide strengthening against bending and shear that is more universal and “cheaper” than a smooth thick film, analogous to profiled plate roofing or walls (e.g., [148]) or curled leaf blades [149]. Such an effect may be especially important for the fragile inner tissues of perianth.

### 5.4. Cuticle Contributes to Heterogeneity in Mechanical Status of the Epidermis Surface

In turgid epidermal cells in situ, relatively thick outer epidermal walls are elastically strained. The amount of the strain differs between consecutive wall layers such that there is an anticlinal, i.e., perpendicular to organ surface, gradient of strain [150,151,152]. When new layers of a growing cell wall are deposited, the existing layers have already been strained (stretched) due to the wall expansion. Therefore, the older wall layers, which are located closer to the organ surface, are most strained while the strain of the recently formed layers, which face the protoplast, is nearly zero. Such an elastic strain gradient could be related to the similar gradient of tensile stress operating in the wall plane (if so, the stress in recently formed layers is nearly zero while older layers are under maximal tensile stress) or the Young’s modulus gradient of the opposite arrow (the younger layers are stiffer than the older ones). Calculations show that in growing cell walls, the elastic strain gradient is most likely related to the similar gradient of tensile stress, while after growth cessation, the modulus gradient may become more important [151]. 

Experiments on fleshy fruits and leaves [63,153], show that the cuticular membranes are elastically strained and most likely prestressed in situ. They shrink after excision from the organ surface followed by isolation from underlying cell walls, especially for the developmental stages characterized by the rapid growth. These cuticular membranes shrink further upon wax extraction [63,64]. Moreover, the anticlinal gradient of elastic strain (referred to as a radial gradient) is predicted for cuticular membranes isolated from the apple fruit surface [153]. Similar to the primary wall layers, this cuticle strain gradient may result from the wall expansion during the cuticle deposition, as long as it is assumed that new cuticle material is deposited preferentially as layers superimposed on the inner surface of the existing cuticle. This postulate is supported by the predominant outward direction of curling (rolling up with older layer inside the roll) of cuticular membranes upon isolation [63,153]. The elastic strain imposed on the cutin scaffold during the expansion of underlying cell wall, is to some extent “fixed” by embedding with intracuticular waxes which leads to plastic cuticle strain and thus reduces a high and potentially catastrophic elastic strain [64,143]. Therefore, the cuticular membrane shrinks gradually upon removal of its consecutive layers (starting from the youngest layer toward the older ones) only on the condition that waxes are extracted from the membrane after the layer removal [153]. 

The strain/stress gradient has been shown for thick cuticular membranes and it is expected only when new material is superimposed as layers and the cuticle is smooth, unlike the folded cuticles of the mature perianth epidermis. Nevertheless, one may predict that in the perianth, the outer periclinal wall of epidermal cells is heterogeneous not only in terms of its properties but also the mechanical state, at least within the primary cell wall layers. One of the consequences of such an anticlinal gradient of elastic strain and possibly also of in-plane stress could be that such cell wall heterogeneity may help a spontaneous movement of cuticle precursors toward the cuticle/organ surface, i.e., to the layers that are more stretched and thus probably easier to penetrate.

## 6. Concluding Remarks and Future Perspective

Cuticle covering perianth organs is special in both its structure and function. Some of these perianth peculiarities are related to the cuticle mechanics. In particular, strengthening of the perianth surface provided by the folded cuticle functionally resembles profiled plates and thus strengthens the organ surface in the way different from thick and smooth cuticles. In some plants, the cuticle covers the petal epidermis that is “punctured” by intercalary air spaces. Then, the cuticle is the only integral continuous layer on the organ surface. The perianth cuticle is distinguished also by those aspects of its mechanics and development that need further studies. The open questions on the cuticle assembly and expansion, and contribution of superimposition and intussusception to these processes, refer to all of the organs. However, more investigations are needed to explain the formation and maintenance of the cuticle folding, which is typical for the perianth epidermis, and also to elucidate mechanical properties and the behavior of the perianth cuticle in situ. Gaps in our knowledge are partly due to technical problems caused by a very small thickness of the perianth cuticle, but modern tools may help to overcome these obstacles. For instance, nanoindentation methods such as atomic force microscopy [140] or cellular force microscopy [154] may help to measure mechanical properties of the cuticle in situ while micromechanical mapping using molecular rotors [155] is a promising method to assess the tension of different cell wall layers. 

Finally, there are important applied aspects of cuticle studies. Technologies are already available to obtain cutin monomers from agro-waste that could be used for bioplastics production [156,157]. Additionally, last but not the least, petal cuticles are explored in biomimetics studies as inspiration for the design of surfaces with unique wetting properties or coloring [66,80,91,158].

## Figures and Tables

**Table 1:** Functions of cuticle in plant organs.

Organ	Function	Species	Source
Flower organs	Prevention of organ fusion	*Arabidopsis thaliana*	[95]
		*Solanum lycopersicon*	[96]
	Protection against insects	*Aristolochia fimbriata*	[97]
Perianth	Control of organ adhesion	*A. thaliana*	[75,85]
	Releasing floral scent	*Clarkia breweri*	[98]
		*Antirrhinum majus*	[99]
Petals	Protection against water loss	*Cosmos bipinnatus*	[100,101]
		*Rosa chinensis* (Movie star, Tineke)	[102]
	Self-cleaning	*Mutisia decurrens*	[103,104]
	Protection against UV radiation		[105]
	Emission of volatiles to attract insect and enhance pollination	*Antirrhinum majus*	[106]
	Facilitation of pollinator attachment		[90]
	Attraction of pollinators by optical effects (structural color, iridescence)		[107,108,109]
Pistil	Control of pollen/pistil interactions	*Brassica, Arabidopsis thaliana*	[82]
		*Oryza sativa*	[110]
Carpel	Prevention of organ fusion	*Catharanthus roseus*	[111]
Fruit	Control of nonstomatal transpiration	*Prunus avium* cv. Sam	[112]
	Control of stomatal transpiration	*Prunus avium* cv. Sam	[113]
	Protection against water loss	*Malus domestica* (Jonagold, Jonagored, Elstar)	[114]
		*Mangifera Indica* cv. Cogshall	[115]
		*Capsicum*	[116]
		*Solanum lycopersicon*	[89,117]
		*Malus domestica*	[118]
	Protection against UV radiation	*Cydonia oblonga*	[81]
	Protection against pathogens	*Solanum lycopersicon*	[87]
	Protection against insects	*Prunus domestica*	[97]
Leaf	Protection against water loss (transpiration barrier)		[80,119]
		*Arabidopsis thaliana*	[120]
		*Zea mays*	[121]
		*Olea europaea*	
		*Triticum aestivum*	
		*Citrus sinensis*	
		*Prunus laurocerasus*	[122]
		*Vanilla planifolia*	
		*Ruellia*	[118]
	Protection against high temperature	*Citrullus colocynthis*	[123]
		*Phoenix dactylifera*	
	Providing superhydrophobic surface	*Salvinia natans*	[80]
		*Aponogeton madagascariensis*	
	Protection against insects	*Arabidopsis thaliana*	[124]
		*Chenopodium album*	[97]
		*Nepenthes albomarginata*	[125]
		*Prunus avium*	
	Protection against pathogens	*Cucumis sativus*	[126]
		*Phaseolus vulgaris*	
		*Arabidopsis thaliana*	[127]
			[128]
		*Ilex aquifolium*	[81]
		*Prunus avium*	
	Protection against UV radiation		[105]
		*Triticum aestivum* cv. Shango	[129]
	Self-cleaning	*Mutisia decurrens*	[103]
		*Nelumbo nucifera*	
		*Colocasia esculenta*	
	Prevention of organ fusion	*Arabidopsis thaliana*	[130,131]
	Triboelectric effect	*Rhododendron*	[30,132]

## Data Availability

Not applicable.

## References

[1] Niklas K.J. (1992). Plant. Biomechanics: An Engineering Approach to Plant Form and Function.

[2] Philippe G., Sorensen I., Jiao C., Sun X., Fei Z., Domozych D.S., Rose J.K. (2020). Cutin and suberin: Assembly and origins of specialized lipidic cell wall scaffolds. Curr. Opin. Plant Biol..

[3] Metcalfe C., Chalk L. (1979). Anatomy of the Dicotyledon, Systematic Anatomy of the Leaf and Stem.

[4] Fernandez V., Guzman-Delgado P., Graca J., Santos S., Gil L. (2016). Cuticle Structure in Relation to Chemical Composition: Re-assessing the Prevailing Model. Front. Plant Sci..

[5] Jeffree C.E. (2006). The fine structure of the plant cuticle. Annu. Plant Rev..

[6] Evert R.F. (2006). Esau’s Plant Anatomy: Meristems, Cells, and Tissues of the Plant Body: Their Structure, Function, and Development.

[7] Guzman P., Fernandez V., Graca J., Cabral V., Kayali N., Khayet M., Gil L. (2014). Chemical and structural analysis of Eucalyptus globulus and *E. camaldulensis* leaf cuticles: A lipidized cell wall region. Front. Plant Sci..

[8] Guzman P., Fernandez V., Garcia M.L., Khayet M., Fernandez A., Gil L. (2014). Localization of polysaccharides in isolated and intact cuticles of eucalypt, poplar and pear leaves by enzyme-gold labelling. Plant. Physiol. Biochem..

[9] Philippe G., Geneix N., Petit J., Guillon F., Sandt C., Rothan C., Lahaye M., Marion D., Bakan B. (2020). Assembly of tomato fruit cuticles: A cross-talk between the cutin polyester and cell wall polysaccharides. New Phytol..

[10] Fich E.A., Segerson N.A., Rose J.K. (2016). The Plant Polyester Cutin: Biosynthesis, Structure, and Biological Roles. Annu. Rev. Plant Biol..

[11] Mazurek S., Garroum I., Daraspe J., de Bellis D., Olsson V., Mucciolo A., Butenko M.A., Humbel B.M., Nawrath C. (2017). Connecting the Molecular Structure of Cutin to Ultrastructure and Physical Properties of the Cuticle in Petals of Arabidopsis. Plant Physiol..

[12] Leide J., Nierop K.G.J., Deininger A.C., Staiger S., Riederer M., de Leeuw J.W. (2020). Leaf cuticle analyses: Implications for the existence of cutan/non-ester cutin and its biosynthetic origin. Ann. Bot..

[13] Boom A., Damsté J.S.S., de Leeuw J.W. (2005). Cutan, a common aliphatic biopolymer in cuticles of drought-adapted plants. Org. Geochem..

[14] Kunst L., Samuels A.L. (2003). Biosynthesis and secretion of plant cuticular wax. Prog. Lipid Res..

[15] Kunst L., Samuels L. (2009). Plant cuticles shine: Advances in wax biosynthesis and export. Curr. Opin. Plant Biol..

[16] Heredia-Guerrero J.A., Benitez J.J., Dominguez E., Bayer I.S., Cingolani R., Athanassiou A., Heredia A. (2014). Infrared and Raman spectroscopic features of plant cuticles: A review. Front. Plant Sci..

[17] Nawrath C. (2006). Unraveling the complex network of cuticular structure and function. Curr. Opin. Plant Biol..

[18] Diarte C., de Souza A.X., Staiger S., Deininger A.C., Bueno A., Burghardt M., Graell J., Riederer M., Lara I., Leide J. (2021). Compositional, structural and functional cuticle analysis of *Prunus laurocerasus* L. sheds light on cuticular barrier plasticity. Plant Physiol. Biochem..

[19] Hunt L., Gray J.E. (2020). How the stomate got his pore: Very long chain fatty acids and a structural cell wall protein sculpt the guard cell outer cuticular ledge. New Phytol..

[20] Takahashi Y., Tsubaki S., Sakamoto M., Watanabe S., Azuma J. (2012). Growth-dependent chemical and mechanical properties of cuticular membranes from leaves of *Sonneratia alba*. Plant Cell Environ..

[21] Shumborski S.J., Samuels A.L., Bird D.A. (2016). Fine structure of the Arabidopsis stem cuticle: Effects of fixation and changes over development. Planta.

[22] Bourgault R., Matschi S., Vasquez M., Qiao P., Sonntag A., Charlebois C., Mohammadi M., Scanlon M.J., Smith L.G., Molina I. (2020). Constructing functional cuticles: Analysis of relationships between cuticle lipid composition, ultrastructure and water barrier function in developing adult maize leaves. Ann. Bot..

[23] Cameron K.D., Teece M.A., Smart L.B. (2006). Increased accumulation of cuticular wax and expression of lipid transfer protein in response to periodic drying events in leaves of tree tobacco. Plant Physiol..

[24] Kosma D.K., Bourdenx B., Bernard A., Parsons E.P., Lu S., Joubes J., Jenks M.A. (2009). The impact of water deficiency on leaf cuticle lipids of Arabidopsis. Plant Physiol..

[25] Seo P.J., Lee S.B., Suh M.C., Park M.J., Go Y.S., Park C.M. (2011). The MYB96 transcription factor regulates cuticular wax biosynthesis under drought conditions in Arabidopsis. Plant Cell.

[26] Amid A., Lytovchenko A., Fernie A.R., Warren G., Thorlby G.J. (2012). The sensitive to freezing3 mutation of *Arabidopsis thaliana* is a cold-sensitive allele of homomeric acetyl-CoA carboxylase that results in cold-induced cuticle deficiencies. J. Exp. Bot..

[27] Bueno A., Sancho-Knapik D., Gil-Pelegrin E., Leide J., Peguero-Pina J.J., Burghardt M., Riederer M. (2019). Cuticular wax coverage and its transpiration barrier properties in *Quercus coccifera* L. leaves: Does the environment matter?. Tree Physiol..

[28] Chen M., Zhu X., Zhang Y., Du Z., Chen X., Kong X., Sun W., Chen C. (2020). Drought stress modify cuticle of tender tea leaf and mature leaf for transpiration barrier enhancement through common and distinct modes. Sci. Rep..

[29] Lewandowska M., Keyl A., Feussner I. (2020). Wax biosynthesis in response to danger: Its regulation upon abiotic and biotic stress. New Phytol..

[30] Bhanot V., Fadanavis S.V., Panwar J. (2021). Revisiting the architecture, biosynthesis and functional aspects of the plant cuticle: There is more scope. Environ. Exp. Bot..

[31] Javelle M., Vernoud V., Rogowsky P.M., Ingram G.C. (2011). Epidermis: The formation and functions of a fundamental plant tissue. New Phytol..

[32] Vrablova M., Vrabl D., Sokolova B., Markova D., Hronkova M. (2020). A modified method for enzymatic isolation of and subsequent wax extraction from *Arabidopsis thaliana* leaf cuticle. Plant Methods.

[33] Suh M.C., Samuels A.L., Jetter R., Kunst L., Pollard M., Ohlrogge J., Beisson F. (2005). Cuticular lipid composition, surface structure, and gene expression in Arabidopsis stem epidermis. Plant Physiol..

[34] Franke R., Briesen I., Wojciechowski T., Faust A., Yephremov A., Nawrath C., Schreiber L. (2005). Apoplastic polyesters in Arabidopsis surface tissues-a typical suberin and a particular cutin. Phytochemistry.

[35] Fabre G., Garroum I., Mazurek S., Daraspe J., Mucciolo A., Sankar M., Humbel B.M., Nawrath C. (2016). The ABCG transporter PEC1/ABCG32 is required for the formation of the developing leaf cuticle in Arabidopsis. New Phytol..

[36] Panikashvili D., Shi J.X., Schreiber L., Aharoni A. (2009). The Arabidopsis DCR encoding a soluble BAHD acyltransferase is required for cutin polyester formation and seed hydration properties. Plant Physiol..

[37] Mazurek S., Mucciolo A., Humbel B.M., Nawrath C. (2013). Transmission Fourier transform infrared microspectroscopy allows simultaneous assessment of cutin and cell-wall polysaccharides of Arabidopsis petals. Plant J..

[38] Bakan B., Marion D. (2017). Assembly of the Cutin Polyester: From Cells to Extracellular Cell Walls. Plants.

[39] Pollard M., Beisson F., Li Y., Ohlrogge J.B. (2008). Building lipid barriers: Biosynthesis of cutin and suberin. Trends Plant Sci..

[40] Dominguez E., Heredia-Guerrero J.A., Heredia A. (2015). Plant cutin genesis: Unanswered questions. Trends Plant Sci..

[41] Cosgrove D.J. (2005). Growth of the plant cell wall. Nat. Rev. Mol. Cell Biol..

[42] Cosgrove D.J. (2014). Re-constructing our models of cellulose and primary cell wall assembly. Curr. Opin. Plant Biol..

[43] Scheller H.V., Ulvskov P. (2010). Hemicelluloses. Annu. Rev. Plant Biol..

[44] Atmodjo M.A., Hao Z., Mohnen D. (2013). Evolving views of pectin biosynthesis. Annu. Rev. Plant Biol..

[45] Pauly M., Gille S., Liu L., Mansoori N., de Souza A., Schultink A., Xiong G. (2013). Hemicellulose biosynthesis. Planta.

[46] Anderson C.T. (2016). We be jammin’: An update on pectin biosynthesis, trafficking and dynamics. J. Exp. Bot..

[47] Lampugnani E.R., Khan G.A., Somssich M., Persson S. (2018). Building a plant cell wall at a glance. J. Cell Sci..

[48] Polko J.K., Kieber J.J. (2019). The Regulation of Cellulose Biosynthesis in Plants. Plant Cell.

[49] Bird D.A. (2008). The role of ABC transporters in cuticular lipid secretion. Plant Sci..

[50] Hurlock A.K., Roston R.L., Wang K., Benning C. (2014). Lipid trafficking in plant cells. Traffic.

[51] Yeats T.H., Rose J.K. (2008). The biochemistry and biology of extracellular plant lipid-transfer proteins (LTPs). Protein Sci..

[52] Benitez J.J., Heredia-Guerrero J.A., Heredia A. (2007). Self-assembly of carboxylic acids and hydroxyl derivatives on mica. A qualitative AFM study. J. Phys. Chem. C.

[53] Heredia-Guerrero J.A., San-Miguel M.A., Sansom M.S., Heredia A., Benitez J.J. (2009). Chemical reactions in 2D: Self-assembly and self-esterification of 9(10),16-dihydroxypalmitic acid on mica surface. Langmuir.

[54] Buschhaus C., Jetter R. (2011). Composition differences between epicuticular and intracuticular wax substructures: How do plants seal their epidermal surfaces?. J. Exp. Bot..

[55] Koch K., Ensikat H.J. (2008). The hydrophobic coatings of plant surfaces: Epicuticular wax crystals and their morphologies, crystallinity and molecular self-assembly. Micron.

[56] Dominguez E., Heredia-Guerrero J.A., Heredia A. (2011). The biophysical design of plant cuticles: An overview. New Phytol..

[57] Stępiński D., Kwiatkowska M., Wojtczak A., Polit J.T., Dominguez E., Heredia A., Popłońska K. (2020). The Role of Cutinsomes in Plant Cuticle Formation. Cells.

[58] Segado P., Heredia-Guerrero J.A., Heredia A., Dominguez E. (2020). Cutinsomes and CUTIN SYNTHASE1 Function Sequentially in Tomato Fruit Cutin Deposition. Plant Physiol..

[59] Khanal B.P., Knoche M. (2017). Mechanical properties of cuticles and their primary determinants. J. Exp. Bot..

[60] Hong L., Brown J., Segerson N.A., Rose J.K., Roeder A.H. (2017). CUTIN SYNTHASE 2 Maintains Progressively Developing Cuticular Ridges in Arabidopsis Sepals. Mol. Plant..

[61] Hepler P.K., Rounds C.M., Winship L.J. (2013). Control of cell wall extensibility during pollen tube growth. Mol. Plant.

[62] Dumais J. (2013). Modes of deformation of walled cells. J. Exp. Bot..

[63] Wiedemann P., Neinhuis C. (1998). Biomechanics of isolated plant cuticles. Bot. Acta.

[64] Lai X., Khanal B.P., Knoche M. (2016). Mismatch between cuticle deposition and area expansion in fruit skins allows potentially catastrophic buildup of elastic strain. Planta.

[65] Koch K., Neinhuis C., Ensikat H.J., Barthlott W. (2004). Self assembly of epicuticular waxes on living plant surfaces imaged by atomic force microscopy (AFM). J. Exp. Bot..

[66] Schulte A.J., Droste D.M., Koch K., Barthlott W. (2011). Hierarchically structured superhydrophobic flowers with low hysteresis of the wild pansy (Viola tricolor)—New design principles for biomimetic materials. Beilstein J. Nanotechnol..

[67] Kay Q.O.N., Daoud H.S., Stirton C.H. (1981). Pigment Distribution, Light-Reflection and Cell Structure in Petals. Bot. J. Linn. Soc..

[68] Ojeda I., Francisco-Ortega J., Cronk Q.C. (2009). Evolution of petal epidermal micromorphology in Leguminosae and its use as a marker of petal identity. Ann. Bot..

[69] Martin C., Glover B.J. (2007). Functional aspects of cell patterning in aerial epidermis. Curr. Opin. Plant Biol..

[70] Quintana A., Albrechtová J., Griesbach R.J., Freyre R. (2007). Anatomical and biochemical studies of anthocyanidins in flowers of *Anagallis monelli* L. (Primulaceae) hybrids. Sci. Hortic. Amst..

[71] Thomas M.M., Rudall P.J., Ellis A.G., Savolainen V., Glover B.J. (2009). Development of a complex floral trait: The pollinator-attracting petal spots of the beetle daisy, *Gorteria diffusa* (Asteraceae). Am. J. Bot..

[72] Koch K., Barthlott W. (2009). Superhydrophobic and superhydrophilic plant surfaces: An inspiration for biomimetic materials. Philos. Trans. A Math. Phys. Eng. Sci..

[73] Martens P. (1933). Recherches sur la cuticule. Protoplasma.

[74] Smyth D.R. (2017). Wrinkles on Sepals: Cuticular Ridges Form when Cuticle Production Outpaces Epidermal Cell Expansion. Mol. Plant.

[75] Shi J.X., Malitsky S., de Oliveira S., Branigan C., Franke R.B., Schreiber L., Aharoni A. (2011). SHINE Transcription Factors Act Redundantly to Pattern the Archetypal Surface of Arabidopsis Flower Organs. PLoS Genet..

[76] Khanal B.P., Grimm E., Finger S., Blume A., Knoche M. (2013). Intracuticular wax fixes and restricts strain in leaf and fruit cuticles. New Phytol..

[77] Kourounioti R.L.A., Band L.R., Fozard J.A., Hampstead A., Lovrics A., Moyroud E., Vignolini S., King J.R., Jensen O.E., Glover B.J. (2013). Buckling as an origin of ordered cuticular patterns in flower petals. J. R. Soc. Interface.

[78] Huang X., Hai Y., Xie W.-H. (2017). Anisotropic cell growth-regulated surface micropatterns in flower petals. Theor. Appl. Mech. Lett..

[79] Dumais J., Shaw S.L., Steele C.R., Long S.R., Ray P.M. (2006). An anisotropic-viscoplastic model of plant cell morphogenesis by tip growth. Int. J. Dev. Biol..

[80] Barthlott W., Mail M., Bhushan B., Koch K. (2017). Plant Surfaces: Structures and Functions for Biomimetic Innovations. Nanomicro Lett..

[81] Krauss P., Markstadter C., Riederer M. (1997). Attenuation of UV radiation by plant cuticles from woody species. Plant Cell Environ..

[82] Aharoni A., Dixit S., Jetter R., Thoenes E., van Arkel G., Pereira A. (2004). The SHINE clade of AP2 domain transcription factors activates wax biosynthesis, alters cuticle properties, and confers drought tolerance when overexpressed in Arabidopsis. Plant Cell.

[83] Bargel H., Neinhuis C. (2005). Tomato (*Lycopersicon esculentum Mill.*) fruit growth and ripening as related to the biomechanical properties of fruit skin and isolated cuticle. J. Exp. Bot..

[84] Serrano M., Coluccia F., Torres M., L’Haridon F., Metraux J.-P. (2014). The cuticle and plant defense to pathogens. Front. Plant Sci..

[85] Sieber P., Schorderet M., Ryser U., Buchala A., Kolattukudy P., Metraux J.P., Nawrath C. (2000). Transgenic Arabidopsis plants expressing a fungal cutinase show alterations in the structure and properties of the cuticle and postgenital organ fusions. Plant Cell.

[86] Li-Beisson Y., Pollard M., Sauveplane V., Pinot F., Ohlrogge J., Beisson F. (2009). Nanoridges that characterize the surface morphology of flowers require the synthesis of cutin polyester. Proc. Natl. Acad. Sci. USA.

[87] Isaacson T., Kosma D.K., Matas A.J., Buda G.J., He Y., Yu B., Pravitasari A., Batteas J.D., Stark R.E., Jenks M.A. (2009). Cutin deficiency in the tomato fruit cuticle consistently affects resistance to microbial infection and biomechanical properties, but not transpirational water loss. Plant J..

[88] Riederer M., Schreiber L. (2001). Protecting against water loss: Analysis of the barrier properties of plant cuticles. J. Exp. Bot..

[89] Leide J., Hildebrandt U., Reussing K., Riederer M., Vogg G. (2007). The developmental pattern of tomato fruit wax accumulation and its impact on cuticular transpiration barrier properties: Effects of a deficiency in a beta-ketoacyl-coenzyme A synthase (LeCER6). Plant Physiol..

[90] Bräuer P., Neinhuis C., Voigt D. (2016). Attachment of honeybees and greenbottle flies to petal surfaces. Arthropod Plant Interact..

[91] Whitney H.M., Federle W. (2013). Biomechanics of plant-insect interactions. Curr. Opin. Plant Biol..

[92] Gorb E.V., Gorb S.N. (2017). Anti-adhesive effects of plant wax coverage on insect attachment. J. Exp. Bot..

[93] Matschi S., Vasquez M.F., Bourgault R., Steinbach P., Chamness J., Kaczmar N., Gore M.A., Molina I., Smith L.G. (2020). Structure-function analysis of the maize bulliform cell cuticle and its potential role in dehydration and leaf rolling. Plant Direct.

[94] Leyton L., Juniper B.E. (1963). Cuticle Structure and Water Relations of Pine Needles. Nature.

[95] Weng H., Molina I., Shockey J., Browse J. (2010). Organ fusion and defective cuticle function in a lacs1 lacs2 double mutant of Arabidopsis. Planta.

[96] Smirnova A., Leide J., Riederer M. (2013). Deficiency in a very-long-chain fatty acid beta-ketoacyl-coenzyme a synthase of tomato impairs microgametogenesis and causes floral organ fusion. Plant Physiol..

[97] Borodich F.M., Gorb E.V., Gorb S.N. (2010). Fracture behaviour of plant epicuticular wax crystals and its role in preventing insect attachment: A theoretical approach. Appl. Phys. A.

[98] Jetter R., Dudareva N., Pichersky E. (2006). Examination of the processes involved in the emission of scent volatiles from flowers. Biology of Floral Scent.

[99] Muhlemann J.K., Klempien A., Dudareva N. (2014). Floral volatiles: From biosynthesis to function. Plant Cell Environ..

[100] Buschhaus C., Hager D., Jetter R. (2015). Wax layers on Cosmos bipinnatus petals contribute unequally to total petal water resistance. Plant Physiol..

[101] Buschhaus C., Peng C., Jetter R. (2013). Very-long-chain 1,2- and 1,3-bifunctional compounds from the cuticular wax of Cosmos bipinnatus petals. Phytochemistry.

[102] Cheng G., Huang H., Zhou L., He S., Zhang Y., Cheng X. (2019). Chemical composition and water permeability of the cuticular wax barrier in rose leaf and petal: A comparative investigation. Plant Physiol. Biochem..

[103] Barthlott W., Neinhuis C. (1997). Purity of the sacred lotus, or escape from contamination in biological surfaces. Planta.

[104] Neinhuis C. (1997). Characterization and Distribution of Water-repellent, Self-cleaning Plant Surfaces. Ann. Bot..

[105] Pfündel E.E., Agati G., Cerovic Z.G., Riederer M., Muller C. (2008). Optical properties of plant surfaces. Annual Plant Reviews.

[106] Goodwin S.M., Kolosova N., Kish C.M., Wood K.V., Dudareva N., Jenks M.A. (2003). Cuticle characteristics and volatile emissions of petals in Antirrhinum majus. Physiol. Plant..

[107] Glover B.J., Whitney H.M. (2010). Structural colour and iridescence in plants: The poorly studied relations of pigment colour. Ann. Bot..

[108] Whitney H.M., Kolle M., Andrew P., Chittka L., Steiner U., Glover B.J. (2009). Floral iridescence, produced by diffractive optics, acts as a cue for animal pollinators. Science.

[109] Whitney H.M., Bennett K.M., Dorling M., Sandbach L., Prince D., Chittka L., Glover B.J. (2011). Why do so many petals have conical epidermal cells?. Ann. Bot..

[110] Chang Z., Chen Z., Yan W., Xie G., Lu J., Wang N., Lu Q., Yao N., Yang G., Xia J. (2016). An ABC transporter, OsABCG26, is required for anther cuticle and pollen exine formation and pollen-pistil interactions in rice. Plant Sci..

[111] Verbeke J.A. (1992). Fusion Events during Floral Morphogenesis. Annu. Rev. Plant Phys..

[112] Knoche M., Peschel S., Hinz M., Bukovac M.J. (2001). Studies on water transport through the sweet cherry fruit surface: II. Conductance of the cuticle in relation to fruit development. Planta.

[113] Peschel S., Beyer M., Knoche M. (2003). Surface characteristics of sweet cherry fruit: Stomata-number, distribution, functionality and surface wetting. Sci. Hortic. Amst..

[114] Veraverbeke E.A., Verboven P., Scheerlinck N., lan Hoang M., Nicolaï B.M. (2003). Determination of the diffusion coefficient of tissue, cuticle, cutin and wax of apple. J. Food Eng..

[115] Lechaudel M., Lopez-Lauri F., Vidal V., Sallanon H., Joas J. (2013). Response of the physiological parameters of mango fruit (transpiration, water relations and antioxidant system) to its light and temperature environment. J. Plant. Physiol..

[116] Parsons E.P., Popopvsky S., Lohrey G.T., Lu S., Alkalai-Tuvia S., Perzelan Y., Paran I., Fallik E., Jenks M.A. (2012). Fruit cuticle lipid composition and fruit post-harvest water loss in an advanced backcross generation of pepper (*Capsicum* sp.). Physiol. Plant.

[117] Vogg G., Fischer S., Leide J., Emmanuel E., Jetter R., Levy A.A., Riederer M. (2004). Tomato fruit cuticular waxes and their effects on transpiration barrier properties: Functional characterization of a mutant deficient in a very-long-chain fatty acid beta-ketoacyl-CoA synthase. J. Exp. Bot..

[118] Schreiber L., Riederer M. (1996). Ecophysiology of cuticular transpiration: Comparative investigation of cuticular water permeability of plant species from different habitats. Oecologia.

[119] Kerstiens G. (1996). Cuticular water permeability and its physiological significance. J. Exp. Bot..

[120] Bessire M., Borel S., Fabre G., Carraca L., Efremova N., Yephremov A., Cao Y., Jetter R., Jacquat A.C., Metraux J.P. (2011). A member of the PLEIOTROPIC DRUG RESISTANCE family of ATP binding cassette transporters is required for the formation of a functional cuticle in Arabidopsis. Plant Cell.

[121] Fernandez V., Bahamonde H.A., Peguero-Pina J.J., Gil-Pelegrin E., Sancho-Knapik D., Gil L., Goldbach H.E., Eichert T. (2017). Physico-chemical properties of plant cuticles and their functional and ecological significance. J. Exp. Bot..

[122] Schreiber L., Bach S., Kirsch T., Knoll D., Schalz K., Riederer M. (1995). A simple photometric device analysing cuticular transport physiology: Surfactant effect on permeability of isolated cuticular membranes of *Prunus laurocerasus* L.. J. Exp. Bot..

[123] Bueno A., Alfarhan A., Arand K., Burghardt M., Deininger A.C., Hedrich R., Leide J., Seufert P., Staiger S., Riederer M. (2019). Effects of temperature on the cuticular transpiration barrier of two desert plants with water-spender and water-saver strategies. J. Exp. Bot..

[124] Heredia A., Heredia-Guerrero J.A., Dominguez E., Benitez J.J. (2009). Cutin synthesis: A slippery paradigm. Biointerphases.

[125] Riedel M., Eichner A., Meimberg H., Jetter R. (2007). Chemical composition of epicuticular wax crystals on the slippery zone in pitchers of five Nepenthes species and hybrids. Planta.

[126] Tenberge K.B. (2007). Morphology and Cellular Organisation in Botrytis Interactions with Plants. Botrytis: Biology, Pathology and Control.

[127] Bessire M., Chassot C., Jacquat A.C., Humphry M., Borel S., Petetot J.M., Metraux J.P., Nawrath C. (2007). A permeable cuticle in Arabidopsis leads to a strong resistance to Botrytis cinerea. EMBO J..

[128] Deising H.B., Werner S., Wernitz M. (2000). The role of fungal appressoria in plant infection. Microbes Infect..

[129] Meyer S., Cartelat A., Moya I., Cerovic Z.G. (2003). UV-induced blue-green and far-red fluorescence along wheat leaves: A potential signature of leaf ageing. J. Exp. Bot..

[130] Iwakawa H., Iwasaki M., Kojima S., Ueno Y., Soma T., Tanaka H., Semiarti E., Machida Y., Machida C. (2007). Expression of the ASYMMETRIC LEAVES2 gene in the adaxial domain of Arabidopsis leaves represses cell proliferation in this domain and is critical for the development of properly expanded leaves. Plant J..

[131] Tanaka T., Tanaka H., Machida C., Watanabe M., Machida Y. (2004). A new method for rapid visualization of defects in leaf cuticle reveals five intrinsic patterns of surface defects in Arabidopsis. Plant J..

[132] Meder F., Must I., Sadeghi A., Mondini A., Filippeschi C., Beccai L., Mattoli V., Pingue P., Mazzolai B. (2018). Energy Conversion at the Cuticle of Living Plants. Adv. Funct. Mater..

[133] Ginzberg I., Stern R.A. (2019). Control of Fruit Cracking by Shaping Skin Traits—Apple as a Model. Crit. Rev. Plant Sci..

[134] Matas A.J., Cobb E.D., Paolillo D.J., Niklas K.J. (2004). Crack resistance in cherry tomato fruit correlates with correlates with cuticular membrane thickness. Hortscience.

[135] Koch K., Bennemann M., Bohn H.F., Albach D.C., Barthlott W. (2013). Surface microstructures of daisy florets (Asteraceae) and characterization of their anisotropic wetting. Bioinspir. Biomim..

[136] Petracek P.D., Bukovac M.J. (1995). Rheological Properties of Enzymatically Isolated Tomato Fruit Cuticle. Plant Physiol..

[137] Burgert I., Fratzl P., Verbelen J.-P., Vissenberg K. (2006). Mechanics of the Expanding Cell Wall. The Expanding Cell.

[138] Roelofsen P.A. (1952). On the Submicroscopic Structure of Cuticular Cell Walls. Acta Bot. Neerl..

[139] Matas A.J., Lopez-Casado G., Cuartero J., Heredia A. (2005). Relative humidity and temperature modify the mechanical properties of isolated tomato fruit cuticles. Am. J. Bot..

[140] Round A.N., Yan B., Dang S., Estephan R., Stark R.E., Batteas J.D. (2000). The influence of water on the nanomechanical behavior of the plant biopolyester cutin as studied by AFM and solid-state NMR. Biophys. J..

[141] Heredia-Guerrero J.A., Guzman-Puyol S., Benitez J.J., Athanassiou A., Heredia A., Dominguez E. (2018). Plant cuticle under global change: Biophysical implications. Glob. Chang. Biol..

[142] Bargel H., Koch K., Cerman Z., Neinhuis C. (2006). Structure-function relationships of the plant cuticle and cuticular waxes—A smart material?. Funct. Plant Biol..

[143] Knoche M., Lang A. (2017). Ongoing Growth Challenges Fruit Skin Integrity. Crit. Rev. Plant Sci..

[144] Onoda Y., Schieving F., Anten N.P. (2015). A novel method of measuring leaf epidermis and mesophyll stiffness shows the ubiquitous nature of the sandwich structure of leaf laminas in broad-leaved angiosperm species. J. Exp. Bot..

[145] Liang H., Mahadevan L. (2009). The shape of a long leaf. Proc. Natl. Acad. Sci. USA.

[146] Hiller G.H. (1884). Untersuchungen über die Epidermis der Blüthenblätter. Jahrb. Wiss. Bot..

[147] Borowska-Wykręt D., Kwiatkowska D., Geitmann A., Gril J. (2018). Folding, Wrinkling, and Buckling in Plant Cell Walls. Plant Biomechanics: From Structure to Function at Multiple Scales.

[148] Wright H.D., Hossain K.M.A. (1997). In-plane shear behaviour of profiled steel sheeting. Thin Walled Struct..

[149] King M.J., Vincent J.F.V., Harris W. (1996). Curling and folding of leaves of monocotyledons—A strategy for structural stiffness. N. Z. J. Bot..

[150] Hejnowicz Z., Borowska-Wykręt D. (2005). Buckling of inner cell wall layers after manipulations to reduce tensile stress: Observations and interpretations for stress transmission. Planta.

[151] Lipowczan M., Borowska-Wykręt D., Natonik-Białoń S., Kwiatkowska D. (2018). Growing cell walls show a gradient of elastic strain across their layers. J. Exp. Bot..

[152] Natonik-Białoń S., Borowska-Wykręt D., Mosca G., Grelowski M., Wrzalik R., Smith R.S., Kwiatkowska D. (2020). Deformation of a cell monolayer due to osmotic treatment: A case study of onion scale epidermis. Botany.

[153] Khanal B.P., Knoche M., Bussler S., Schluter O. (2014). Evidence for a radial strain gradient in apple fruit cuticles. Planta.

[154] Routier-Kierzkowska A.L., Weber A., Kochova P., Felekis D., Nelson B.J., Kuhlemeier C., Smith R.S. (2012). Cellular force microscopy for in vivo measurements of plant tissue mechanics. Plant Physiol..

[155] Michels L., Gorelova V., Harnvanichvech Y., Borst J.W., Albada B., Weijers D., Sprakel J. (2020). Complete microviscosity maps of living plant cells and tissues with a toolbox of targeting mechanoprobes. Proc. Natl. Acad. Sci. USA.

[156] Heredia-Guerrero J.A., Heredia A., Dominguez E., Cingolani R., Bayer I.S., Athanassiou A., Benitez J.J. (2017). Cutin from agro-waste as a raw material for the production of bioplastics. J. Exp. Bot..

[157] Benitez J.J., Osbild S., Guzman-Puyol S., Heredia A., Heredia-Guerrero J.A. (2020). Bio-Based Coatings for Food Metal Packaging Inspired in Biopolyester Plant Cutin. Polymers.

[158] Sun J., Bhushan B., Tong J. (2013). Structural coloration in nature. RSC Adv..

